# Local Pain Dynamics during Constant Exhaustive Exercise

**DOI:** 10.1371/journal.pone.0137895

**Published:** 2015-09-30

**Authors:** Agne Slapsinskaite, Selen Razon, Natàlia Balagué Serre, Robert Hristovski, Gershon Tenenbaum

**Affiliations:** 1 Department of Health and Applied Science, INEFC University of Barcelona, Barcelona, Spain; 2 Department of Physical Education, Ball State University, Muncie, Indiana, United States of America; 3 Department of Physical Education, Sport and Health, Ss. Cyril and Methodius University, Skopje, Macedonia; 4 Department of Educational Psychology and Learning Systems, Florida State University, Tallahassee, Florida, United States of America; Texas Tech University Health Science Centers, UNITED STATES

## Abstract

The purpose of this study was to delineate the topological dynamics of pain and discomfort during constant exercise performed until volitional exhaustion. Eleven physical education students were tested while cycling and running at a “hard” intensity level (e.g., corresponding to Borg’s RPE (6–20) = 15). During the tests, participants reported their discomfort and pain on a body map every 15s. “Time on task” for each participant was divided into five equal non-overlapping temporal windows within which their ratings were considered for analysis. The analyses revealed that the number of body locations with perceived pain and discomfort increased throughout the five temporal windows until reaching the mean (± SE) values of 4.2 ± 0.7 and 4.1 ± 0.6 in cycling and running, respectively. The dominant locations included the quadriceps and hamstrings during cycling and quadriceps and chest during running. In conclusion, pain seemed to spread throughout the body during constant cycling and running performed up to volitional exhaustion with differences between cycling and running in the upper body but not in the lower body dynamics.

## Introduction

People experience local discomfort and exertive pain during intense endurance exercise [1,2]. Previous studies have focused on the impact of exertive pain on effort perception, and time to volitional exhaustion [3,4], and investigated the dynamics of its intensity over extended bouts of exercise [5,6]. Nevertheless, no studies have explored the evolution of exertive pain and its spatiotemporal topology dynamics during exercise.

Exertive pain is defined as a subjective state or feeling associated with tiredness, soreness and numbness [7]. Exertive pain is different from perceived exertion in that the latter is the subjective feeling of heaviness and strain, which stems from physical effort [8]. Athletes competing in aerobic activities refer to “discomfort” in relation to symptoms emanating from legs, respiratory system, proprioceptive system, head, and abdomen [9]. Others have also referred to discomfort in exercising peripheral skeletal muscles as a common limit to different strenuous tasks [10,11] or as a limiting factor to exercise in forms of breathlessness and leg fatigue [3]. The latter is consistent with the notion that there are differentiated rating of perceived exertion (RPE) for legs (RPE-Legs) and chest (RPE-Chest) [12]. Specifically, during a cycling ramp type protocol, RPE-Legs and RPE-Chest have shown to contribute equally to the RPE-Overall, and thus to the whole-body sensory integration process [13].

The perception of discomfort and pain vary inter-individually [14,15]. However, gender differences across all pain modalities have not been supported during exercise [16]. Interestingly, individuals with similar patterns of activation of the primary somatosensory cortex (SI), anterior cingulate cortex (ACC), and prefrontal cortex (PFC) provide similar subjective reports of pain magnitude [17]. This implies that an individual’s conscious experience of pain can be accurately captured via introspection [17]. The correlations between brain imaging and pain ratings also provide some external validation of self-report for pain monitoring [18].

A number of introspective methods are used to measure perceived exertion (e.g., RPE (6–20) Borg scale), pain intensity (e.g., numerical pain rating scales) and pain location (e.g., pain drawings, body maps, or manikins) [19]. Of these, pain drawings have been used to measure widespread pain in static conditions of self-report in long-term intervals (e.g., 6 weeks, 6 months, or 1 year) [19,20]. Nevertheless, the evaluation of dynamical changes at short-term intervals is yet to be considered. To date, only the intensity of exertive pain, but not its spatiotemporal topology and spreading patterns, has been studied. Consequently, there is a lack of knowledge about the dynamics of pain and discomfort during constant exercise performed until volitional exhaustion. Additionally, exercise protocols up to date have been either incremental [5] or self-paced [21], but not constant. Consequently, a constant exercise protocol with continuous and steady effort output requirements may be particularly beneficial for investigating the dynamics of pain spread across the body.

The nonlinear dynamical systems theory (NDST) along with the self-monitoring and self-reporting procedures were used for studying the dynamics of perceived exertion and attention allocation during constant running and cycling tasks performed until volitional exhaustion [22–24]. Findings from these studies revealed a non-proportional effect of effort accumulation on thought dynamics and perceived exertion shifts (PES). Moreover, previous accounts of exertive pain and discomfort have neither considered nominal changes in locations of topologically defined body locations nor have they entirely detailed their effect on volitional exhaustion [25]. The purpose of the present study was to delineate the spatiotemporal topology dynamics of pain and discomfort during constant exercise performed until volitional exhaustion. Specifically, we hypothesized that the number of body locations with discomfort and pain would increase while performing running and cycling tasks as a function of “time on task” and that the lower and upper body dynamics would depend on the type of exercise task.

## Method

### Participants

To determine the sample size for this study a power analysis was conducted using G*Power 3.1 [26]. In similar studies of thought processes larger effect sizes have been reported [23]. Thus, using an effect size of d = 1.0, α < 0.05, power (1—β) = 0.80, a sample size of N = 10 emerged. Accordingly, eleven European descent physical education students (7 males and 4 females, M = 20.83 years old, SE = ± 1.33, and BMI = 22.94, SE = ± 2.03) were recruited for this study. Participants had no sport specialization but were engaged in a wide range of aerobic activities at least three times a week. Inclusion criterion for the study was the absence of chronic pain and injuries.

### Intervention and Procedure

One week prior to the experiment, participants completed a baseline incremental cycling and running test to determine the workload and velocity corresponding to their individual RPE (6–20 Borg’s scale) = 15 (i.e., hard) [8]. During this visit, participants were also familiarized with the use of body map and the reporting procedures (see familiarization). In the second and third weeks, participants performed the experimental constant-power cycling and constant velocity running tasks, respectively, in a counter-balanced order. Participants signed an informed consent prior to taking part in the study. All experimental procedures were approved by the local ethics committee of Catalan Sport Council of Catalonia (Consell Català de l’Esport de la Generalitat de Catalunya, Comitè d'ètica registration number 072015CEICEGC) and were carried out according to the Helsinki Declaration.

#### Discomfort and pain monitoring

**Fig 1:**
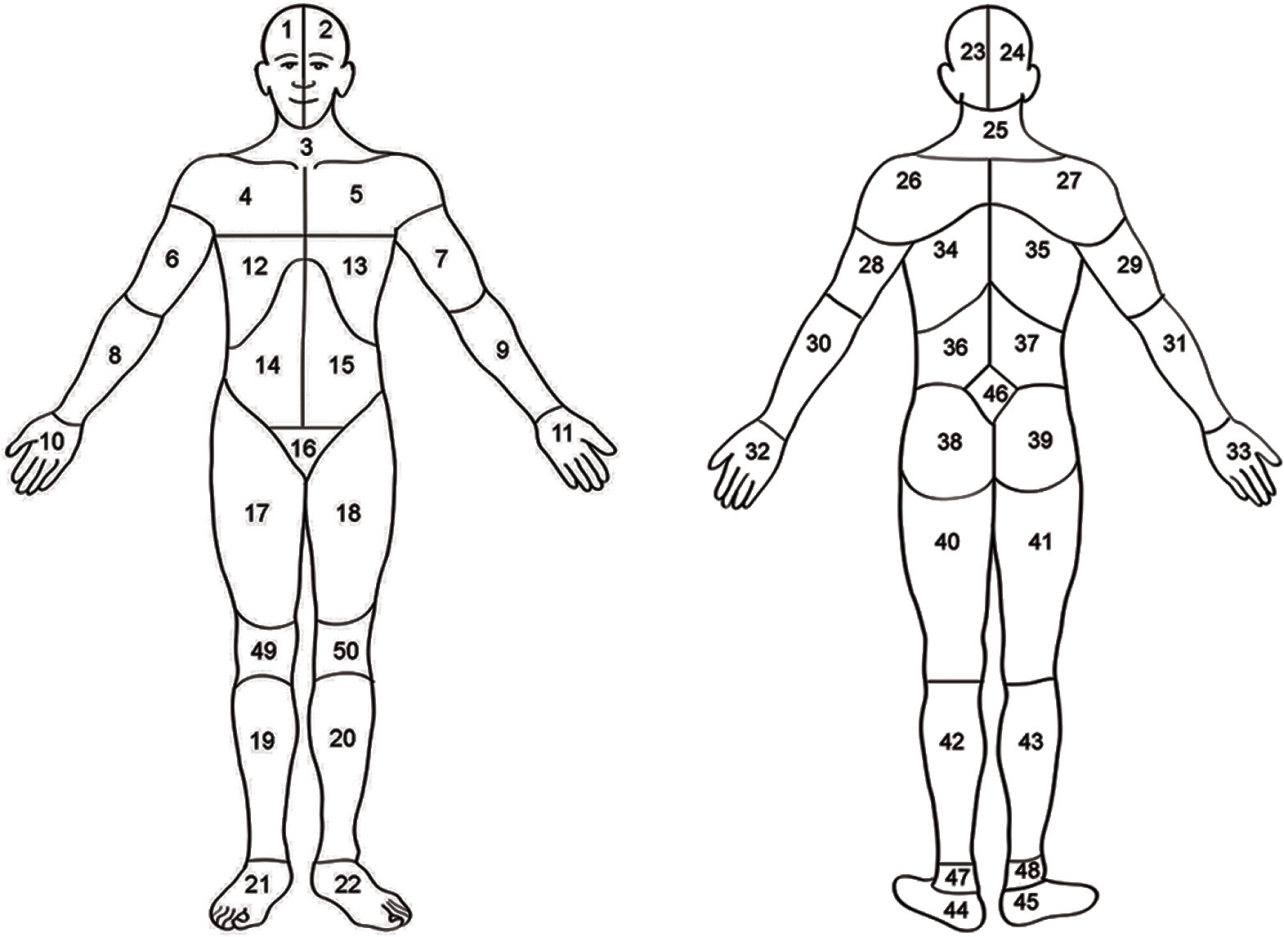
Body map. Head (area 1, 2, 23, or 24); neck (area 3 or 25), shoulders (area 4, 5, 26, 27); arms (area 6, 7, 8, 9, 28, 29, 30 or 31); hand (area 10, 11, 32, 33); ribs or chest (area 12 or 13); abdomen (area 14 or 15), back (area 34, 35, 36, 37), buttocks or hips (area 38 or 39); genitalia (area 16), legs (area 17, 18, 19, 20, 40, 41, 42 or 43); feet (area 21, 22, 44 or 45). Adapted from Margolis, Tait, & Krause (1986).

Throughout the exercise protocol, every 15s, upon the researcher’s prompt, participants reported body locations with discomfort and pain, using a body map (see Fig 1) [27]. The rational for selecting this reporting strategy was to provide ample data points and limiting the potentially deleterious effects of reporting somatic sensations while constantly keeping an internal focus of attention [28]. For the purposes of this assessment, the instructions given to the participants were as follows:

“*When prompted*, *we ask you to report the locations of discomfort and pain (if you feel it*, *independently of its magnitude) using the numbers on the body map placed in front of you*.*”*

#### Familiarization procedures and baseline tests

All the participants have been previously familiarized with the RPE (6–20) Borg scale and have used it during incremental exercises at least two times during the last two months. The RPE (6–20) Borg scale and its original rating instructions [8], as well as the body map were handed out and verbally explained to participants prior to the baseline test and experimental procedures.

The baseline incremental cycling test and the constant-power cycling task were performed on a cycle ergometer (Sport Excalibur 925900) with saddle and handlebar specifications adjusted to fit the preference of each participant. The cycling test started with a 2min rest following an initial load of 20W (for females) and 25W (for males). The load was increased by 20W/min (for females) and 25W/min (for males) until participants reported RPE = 15 keeping the required cadence (70rpm). At this intensity (RPE = 15) participants kept pedalling for 2min while reporting their discomfort and pain (see above). During the last 10s of every imposed workload increment, participants were asked to self-monitor and report verbally their RPE on the RPE (6–20) Borg scale with the corresponding anchors placed in front of them at eye level. The workload values corresponding to RPE = 15 were recorded for each participant. If an identical RPE value was reported over two consecutive increments the highest workload value was registered. When the clamped value of RPE = 15 was not reported the value corresponding to the same Borg’s RPE (6–20 anchor RPE = 14) was recorded.

The baseline incremental running test (HP Cosmos treadmill) consisted of an initial velocity of 5km/h followed by increases of 1km/h per minute (for males) and 0.5km/h per minute (for females) until they reached a velocity corresponding to RPE = 15. At this intensity participants kept running for 2min while reporting locations with pain and discomfort (see above). Identical self-monitoring and RPE reporting and recording procedures were followed as the cycling protocol.

#### Constant-power cycling task

The task included two consecutive parts. An initial incremental warm-up (same procedure as in the corresponding baseline test) and a constant-power cycling task were performed up to volitional exhaustion. The constant-power cycling began when participants reached RPE = 15 during the incremental warm-up and lasted up to volitional exhaustion when participants could no longer maintain the fixed pedalling cadence for 5 consecutive seconds while in the sitting position. The RPE (6–20) Borg scale was placed in front of the participants at eye level during the warm up phase and was replaced by the body map during the constant protocol phase. Only the data from participants reporting RPE = 15 at the same target workload obtained during the baseline incremental cycling test were included in the analysis.

#### Constant velocity running task

The task included two consecutive parts: an incremental warm-up (same procedure as in the corresponding baseline test) and a constant velocity run performed up to volitional exhaustion. The constant velocity run began when participants reached RPE = 15 during the incremental warm-up and lasted up to volitional exhaustion when they could no longer maintain the imposed velocity. The RPE (6–20) Borg scale was placed in front of the participants at eye level during the warming up and was replaced by the body map during the constant protocol. Only the data from participants reporting RPE = 15 at the target velocity obtained during the baseline incremental running test were included in the analysis.

Once the test began, participants performed the cycling/running tasks without any additional communication except for the researcher’s prompts for reporting locations. All trials were video recorded to cross validate the accuracy of the collected data. Upon task completion, using a 11 point Likert-type scale with anchors ranging from 0 (*not at all*) to 10 (*greatly*), participants answered two questions to report their adherence to: (a) the cycling/running task (i.e., “*Have you pedalled/ran as long as you can*, *achieving your exhaustion point*?”), and (b) the self-monitoring and reporting procedure (i.e., “*Have you reported all the changes in your discomfort and pain locations when required*?”).

### Data Analysis

The reported number of locations with pain and discomfort while performing the tasks were plotted for each participant and the locations were anatomically grouped for lower and upper body areas. Each time series was divided into five non-overlapping temporal windows (time to volitional exhaustion of the participant/5). Mean value of the number of locations with discomfort and pain in total was computed for each time window. The probability of each pain location (according to the body map) was computed for each temporal window. A Friedman ANOVA was used to analyse the number of locations variance over time. Effect sizes (Cohen’s d) were calculated to determine means’ differences at p < 0.05. Mann Whitney U matched pairs test was used to contrast the pain dynamics in lower and upper body during both tasks.

## Results

Time to exhaustion during constant cycling at 195 ± 14.3W was 915 ± 62s and constant running at 15.36 ± 0.7km/h was 738 ± 52s. The number of locations with pain and discomfort increased throughout the five temporal windows until reaching the values of 4.2 ± 0.7 for cycling and 4.1 ± 0.6 for running. The Friedman ANOVA revealed a significant effect of time for the total number of locations with discomfort and pain χ^2^ (11,4) = 14.08, p = 0.007 in cycling, and χ^2^ (11,4) = 26.15 p < 0.001 in running. Cohen’s d coefficients between the time windows were as follows: 1^st^ and 3^th^ (-2.95 and -2.19), 1^st^ and 5^th^ (-4.13 and -2.2).

**Fig 2:**
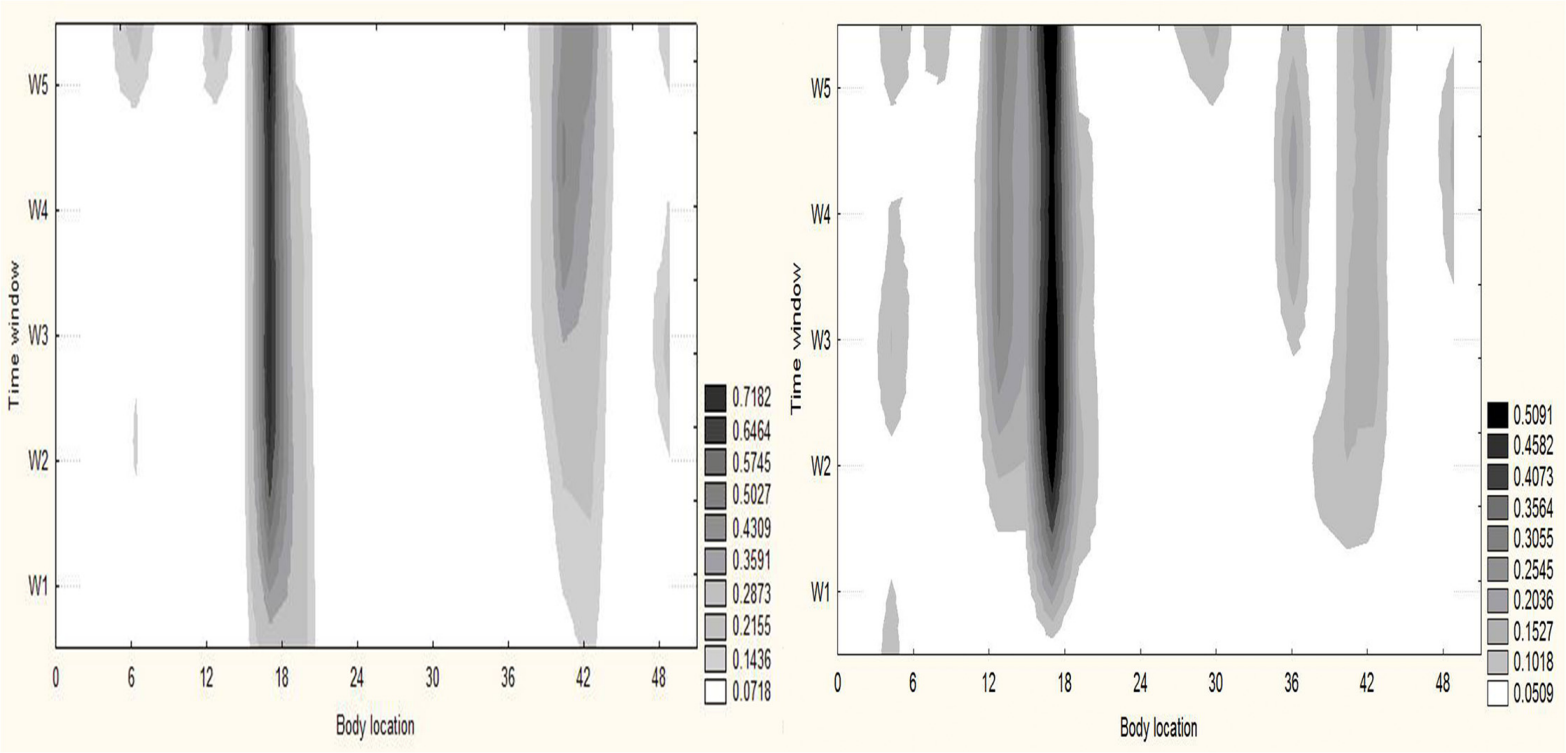
Locations with pain and/or discomfort. The group pulled probabilities of locations with pain or discomfort during cycling (left) and running (right) tasks in 5 temporal windows in a given sample (n = 11). As time on task increases (vertical axis) the number of locations and the probability of experiencing pain and discomfort at selective locations also increase (darker shades of grey) on average. Legend: the probability of experiencing discomfort and pain.

Fig 2 illustrates the frequencies of body locations with pain over time; the darker regions show higher frequencies. The dominant locations of pain and discomfort during both tasks were the quadriceps muscle. At the termination of the exercise task, the dominant locations included the quadriceps and hamstrings in cycling and the quadriceps and chest in running.

The Mann Whitney U test indicated a significant difference only for the upper body locations between the cycling and running tasks in the 2^nd^ (U(20) = 20, p = 0.003), 3^rd^ (U(20) = 25, p = 0.016) and 4^th^ (U(20) = 28.5, p = 0.026) temporal windows.

## Discussion

The main finding of this study revealed that the number of body locations where discomfort and pain are reported during constant cycling and running tasks until volitional exhaustion increased over time. While the lower-body locations increased significantly during both cycling and running, upper-body locations increased significantly only during running.

These findings are consistent with previous research related to the discomfort and exertive pain intensity during incremental and self-paced exhaustive exercise. A linear co-variation between the muscle pain intensity and power output in cycling has been found with lowest pain ratings at the lowest exercise intensity and highest pain ratings at the highest exercise intensity [5]. Increasing ratings have been also indicated in self-paced cycling [21]. Importantly, the present findings also suggest that not only the pain intensity [29] but also the number of pain locations and their frequencies raised while approaching volitional exhaustion. It is evident that the pain threshold (i.e., the point at which an individual perceives a stimulus to be painful), and the pain tolerance (i.e., the point at which an individual is not willing to endure further noxious stimuli) change with the time on task and effort accumulation [30]. However, further research is needed to define the relationship between exercise workload and topology of exertive pain, and extend our findings by exploring the spread patterns of the pain locations. As local pain is task-dependent it will be particularly interesting to test the pain dynamics in incremental or graded exercise protocols. In this study a constant-power instead of incremental-power exercise was chosen a) to prevent from faster task disengagement, especially in less fit participants; b) to avoid changes of the afferent sensory information due to power intensity changes.

Previous studies examining the dynamics of attention focus and effort perception during constant cycling and running until volitional exhaustion have also revealed an effect of time on task (i.e., effort accumulation) on attention allocation and perceived exertion [22–24]. Results from these studies suggested qualitative changes from dissociative to dominantly associative focus of attention, and from fluctuating to non-fluctuating perceived exertion shifts while cycling continuously at RPE = 15 and while running at 80% of maximal heart rate [22–24]. The current findings indicate that as a result of the increased effort output, pain spreads throughout the body. Some have attributed these phenomena to the progressive global instability occurring over the neuromuscular axis that characterizes the exercise-induced fatigue. Specifically, this instability, involving attention and motivational control loops was thought to ultimately lead to task disengagement [31]. Additional inquiries need to discern if the spread patterns of exertive pain and discomfort are subject to qualitative changes, i.e., whether the number of locations increases by adding new locations (“and” logic), shifting between existing locations (“or” logic) or a combination of both. Understanding these qualitative dynamics can facilitate a higher fit between the present results and those previously related to attention focus and the PES.

To better interpret these findings, two limitations should be considered, in the present study: (a) the intensity of discomfort and exertive pain were not measured, and (b) the sample was limited to physically active individuals. Hence, the current findings do not speak to the intensity of pain and/or its dynamics throughout exercise. Additionally, the implications associated with these findings may not hold true for those who are sedentary or highly trained. In conclusion, although different local patterns are observed in cycling and running, pain appears to spread throughout the body as the effort output and time on task increase. These results are pioneer in that they offer an initial account of the spatiotemporal topology of pain spread during constant exhaustive exercise and thereby respond to calls for further studying exertive pain and its tolerance, as well as performance and regulation of exercise in ‘normal’ samples [29,32].
